# Victim identifiability, number of victims, and unit asking in charitable giving

**DOI:** 10.1371/journal.pone.0300863

**Published:** 2024-03-28

**Authors:** Hajdi Moche, Hulda Karlsson, Daniel Västfjäll

**Affiliations:** 1 Department of Behavioral Sciences and Learning, Linköping University, Linköping, Sweden; 2 JEDI-Lab, Department of Behavioral Sciences and Learning, Linköping University, Linköping, Sweden; 3 Decision Research, Eugene, OR, United States of America; University of Valencia: Universitat de Valencia, SPAIN

## Abstract

This study examines the identifiable victim effect (being more willing to help an identified victim than an unidentified), the singularity effect (i.e., being more willing to help a single identified victim than a group of identified victims), and unit asking (first asking donors for their willingness to donate for one unit and then asking for donations for multiple units) in charitable giving. In five studies (*N* = 7996), we vary the level of identifiability, singularity, and group size. We find that unit asking is making people more sensitive to the number of people in need. Further, while the level of identifiability influences affective reactions, this effect does not extend to donations and, thus, is not affected by unit asking. We do, however, find an “emotion asking effect” where asking donors to rate their affect before donating increase donation levels (compared to donors asked to rate affect after). Emotion asking was attenuated when combined with unit asking.

## Introduction

When do people become more and when do they become less sensitive to the scope of suffering in helping contexts? Ample research shows that positive and negative emotion/affect is one of the cues donors use when faced with an opportunity to help others [1, 2]. Helping others is personally rewarding–creating a “warm glow” [3, 4], and studies employing both correlational and experimental methods find both that happier people give more to charity and that giving more makes people happier, in a circular or mutually reinforcing relationship [5–7]. However, while such emotional reactions can motivate charitable behavior, they may also create less sensitivity to scope. The affective system is an on/off system driven by images and narratives and is relatively insensitive to numbers [8, 9]. Thus, when people respond to large magnitudes of lives at risk by relying on their affective reactions, their response will not scale with the number of people at risk [10, 11].

Two helping effects previously found in the literature demonstrate both the motivating power of affect as well as insensitivity to numbers: The identifiable victim effect and the singularity effect.

The identifiable victim effect (IVE) entails an increased willingness to help if the appeal has an identified victim (e.g., with a photo, name, story) compared to when there is no identified victim(s) [12–17]. The IVE appears to be driven by affect, where both self-reported feelings (e.g., distress, sympathy) and measures of brain activity associated with affect tend to be larger for identified than unidentified victims [12, 18, 19].

The singularity effect, on the other hand, is people’s tendency to help a single identified person in need more often than several identified people who are experiencing the same need, even when the amount needed to save one victim and several victims are kept constant [14, 20–22]. The singularity effect is also driven by affect; people feel stronger positive affect (assessed both with self-report and physiological measures), distress, and warm glow for one than for many [21, 23]. Further, the singularity effect is related to compassion fade, which is the tendency for helping intent to decrease as the number of people in need increases [24]. Compassion fade is also partly driven by affect—people experience decreased positive affect (and perceived impact) of helping as the size of the group increases [24].

Thus, both IVE and the singularity effect can be seen as instances where affect increases motivation to help a single identified individual [25], but at the same time an unidentified individual or a group of individuals fail to evoke such affective reactions, thereby reducing the help given.

Recent research has shown that the IVE and singularity effects mostly occur when donors evaluate each option separately (e.g., either evaluate 1 or 8 persons). However, in joint evaluation (e.g., evaluating 1 and 8 persons simultaneously), donors typically display a preference for helping more individuals [14, 26]. Based on this, Hsee et al. [27] proposed a “unit asking” method. In their first study, this method entailed that people were asked to donate to help poor children by first indicating how much they hypothetically would donate to a single child (i.e., a unit) before being asked how much to donate to the whole group of children. This unit asking (UA) method was compared to a control condition, where participants only were asked how much they would want to donate to the whole group of poor children. The results showed that the amount donated to the group of children was significantly higher in the UA condition than in the control condition [27].

While Hsee and colleagues [27] did not find evidence for complete scope sensitivity (i.e., a perfect linear valuation, reflecting that N lives is N times the value of 1 life), they found that UA made people more scope consistent (i.e., participants were consistent in valuing multiple lives more than one life). The UA effect has also been replicated and extended to other domains (e.g., saving animals) [28].

In the original UA study [27] the donation appeal with the poor kindergarten children included a picture of a child, depicting one of the poor children (study 1; this was also the case in the replication study by Karlsson et al. [28]). When a picture is added to a charity appeal like this, it increases the level of identifiability. This involves adding information that identifies the people in need as individuals (often done by adding a picture, name, age, and a short story of a person’s plight) [12, 15], presumably leading to stronger affect. It may even be argued that the UA effect utilizes the IVE and the singularity effect by letting participants first valuate–and thereby anchor—on a single, identified child. The single identified child can make people react with stronger affective reactions and increased willingness to help [14, 21]. Therefore, one possibility is that the use of a picture depicting a single child in the original UA study can have given rise to an IVE and singularity effect and thereby, boosted or even caused the UA effect. However, Hsee et al. [27] claimed that the IVE was unrelated to the UA effect. In the discussion of the original paper, the authors do not in detail report but briefly mention the result of a 2(method: control vs. unit asking) × 2(victim identifiability: high vs. low) factorial study which they conducted to rule out identifiability as an explanation. In this, Hsee et al. describe that they found main effects of identifiability (more giving to identified victims) and unit asking, but no interaction effect. Thus, this result would suggest that even though UA relies on IVE and singularity who, in turn, both are driven by affect, it is unclear what role affect (if any) has in a joint evaluation setting like UA. Thus, there is a need to replicate the Hsee et al. [27] finding and extend it with novel experimental manipulations and measures of affect. Is the affect associated with single identified lives reduced so that donors become more sensitive to scope, but overall give less? Or is the affect associated with a single victim increasing overall levels of giving?

In the present research, we systematically combine the IVE, singularity effect, and unit asking. As outlined above, these three effects are interrelated but have so far not been studied simultaneously or systematically. By doing this, we will be able to better assess the predictions that 1) given that more identifying information leads to stronger affect, donors will express a higher Willingness-to-donate (WTD) than in conditions with less identifying information [12, 18, 19] (thus, affect could impact WTD in an additive way increasing overall levels of helping both for the single and the many recipients, and 2) that affect is reduced by unit asking: A recent study by Moche et al. [22] showed that making people scope sensitive through deliberation interventions came at the cost of reducing the giving to a single identified victim, but not increasing giving to the many. UA may have a similar effect since it relies on joint evaluation and people’s wish to be coherent. Relatedly, Ritov and Baron [29] showed that presenting options in a joint (vs. separate) presentation mode decreased reliance on affect in evaluations. Thus, it is possible that the UA may reduce affect (or the impact of affect on WTD) for the single victim. It may also be that the affect associated with single identified victim only increases giving to the one, but not scale to the many [30–32].

We examine these two predictions in a series of studies (see Table 1 for an overview) where we rely on systematic experimental manipulations of victim identifiability (studies 1–4) and number of victims (Study 5) combined with UA. In three studies (studies 1, 2, and 4), we vary levels of identifiability by removing/adding a picture of a child and a personalized story of the child’s plight to see its effect on the UA method. In Study 3, we examine if the effect of identifiability can be amplified with mental imagery as well as examine the effects of asking for ratings of affect or donations first (also done in Study 4). In Study 5, we vary if the identified unit (which participants in the UA method will hypothetically donate to first) is a single child or five children, and how it influences if the whole group to be helped is rather small (20 children) or big (200 children).

**Table 1:** Overview of studies 1–5; description of design, dependent variables, research gap addressed and main findings.

Study	Effect	Experimental manipulation	Dependent variable	Research gap	Main findings
1*	UA	Control vs UA for one unit (i.e., 1 child)	WTD	The effect of identifiability on UA	*WTD*: Sig UA; No IVE; No UA x IVE
	IVE	No picture vs a picture of 1 child			
2a*	UA	Same as study 1	WTD	*Same as study 1* Explored 3 levels of identifiability, and emotion measures	*WTD*: Sig UA; No IVE; No UA x IVE
	IVE	No picture vs a picture of 1 child vs a picture of 1 child and verbal description			
2b	UA	Same as study 1	Emotion		*Emotion;* No UA; Sig IVE; No UA x IVE
	IVE	*Same as study 2a*			
3*	IVE	No picture vs a picture of 1 child (+ mental imagery) vs a picture of 1 child and verbal description (+ mental imagery)	WTD Emotion	Increasing the affective impact of the material to identify IVE, with the aim of using that material to test research question form study 1.	*WTD*: No IVE; Sig EA
	EA	Emotion measures vs WTD measures first			*Emotion*: Sig IVE; Sig EA
4*	UA	Same as study 1	WTD Emotion	*Same as study 1* Adapting existing material, that have previously resulted in an IVE, for testing the main research question. Replicating the EA effect found in study 3.	*WTD*: Sig UA; Sig -IVE; Sig UA x EA
	IVE	Verbal description of the hospitals work vs personal letter from parents to a child who decided in cancer			*Emotion*: No UA; Sig IVE; Sig UA x IVE
	EA	*Same as study 3*			
5	UA	*Same as study 1*	WTD	How does group size and unit size affect the unit asking effect?	*WTD*: Sig UA; No SA; Sig CF; Sig UA x SA; Sig UA x CF
	SA	Unit size was either 1 or 5 children			
	CF	Donation recipient were 20 vs 200 children			

*Preregistered. Scenario: Study 1, 2a, 2b, 3, 5 = Christmas gifts to children at kindergarten (Hsee et al., 2013); Study 4 = Support childhood cancer treatment (Erlandsson et al., 2015). Sig = *p* < .05. WTD = Willingness to donate, all WTD were hypothetical. UA = Unit Asking, IVE = Identifiable victim effect -IVE = reversed IVE, EA = Emotion Asking, SE = Singularity effect, CF = Compassion fade. Emotion = Personal distress; Sympathy, Distress; Empathic concern; Positive feelings; Negative feelings; Donation feelings.

## Studies 1 and 2 (IVE and UA)

### Aim and hypotheses

Study 1 and 2 aims to investigate how the level of identifiability relates to UA in charitable giving in two ways: including or not including a picture of a child (Study 1—a replication of the results mentioned in Hsee et al. [27]) and increasing the level of vivid and personalized information about one child (Study 2). Our Study 1 is a replication of the results mentioned in Hsee et al. [27]. Even though those results suggested that identifiability did not modify UA, we see several reasons why the IVE is theoretically interesting to study in the context of UA:

The level of identifiability may either heighten or reduce the effect of UA on donations. Identifiability levels may heighten it because the IVE might increase the amount donated to one unit and subsequently, the amount to the group due to a form of anchoring or coherent arbitrariness [33]. But it can also reduce the UA effect since identifiability increases affect-richness in the donation decision, which in turn can lead to less scope-sensitive donations [9]. In line with previous studies [27, 28], we hypothesize that we will find a UA effect in both identified and non-identified charity appeals. Given these theoretical possibilities, and that the study reported by Hsee et al. [27] only is mentioned briefly and without detail in the original article, we think replicating this finding and allowing different predictions than the original study is in order.

## Study 1

Study 1 was pre-registered. The pre-registration can be found at https://osf.io/ukqs8/ Data sets for Study 1 and for the other studies in this paper are openly available and can be found in this link as well, along with the S1 Appendix. For each study, we report how we determined our sample size, all data exclusions, all manipulations, and all measures.

### Method

#### Participants

A total of 1,025 participants were recruited through Prolific at the end of summer 2020. For all the experiments in this paper the following applies; Informed, written consent was obtained from all participants. The authors had no access to information that could identify the participants during or after the data collection. The sample size was determined by aiming to collect at least 200–250 participants in each condition. To remain close to the original study by Hsee et al. [27], we chose all-American samples. For study 1, one did not consent and 27 participants failed the attention check, so the total sample consisted of 997 participants (47.3% women, *M*_*age*_ = 34.4, *SD*_*age*_ = 11.5). The online experiment took approximately five minutes to complete, and all participants received a small monetary compensation for their participation–which is true for all studies in this paper.

Ethics statement. Informed consent was collected from all participants. According to guidelines from the Swedish research council concerning the Ethical Review of Research Involving Humans (SFS 2003:460), approval from an ethics committee is waived for behavioral research such as this study.

#### Design

The study had a 2 (Manipulation group: control vs. unit asking) × 2 (Identifiability level: non-identified vs. identified) between-subject design. Participants were randomly assigned to one of the four conditions. The dependent variable was willingness-to-donate (WTD) to the 20 children. As in the original study by Hsee et al. [27], all donations were hypothetical.

#### Procedure

Initially, participants read a short description of the study, consented to participate, and answered an attention check and demographic questions. Following, participants were presented with a scenario that was identical to study 1 in Hsee et al. [27]. Participants were asked to imagine that Christmas was around the corner and that they had received an email from a kindergarten principal. They read that the kindergarten asked for donations to buy Christmas gifts for 20 children from low-income families. Participants in the non-identified condition read this information only, with no picture included in the appeal. For participants in the identified condition, the text also included a picture of a small girl, who was described as one of the children from the low-income families. This latter condition was an exact replication of study 1 by Hsee et al. [27]. Thus, the difference between the conditions was whether there was a picture of a child included in the charity request. Participants in the control condition were asked to indicate their WTD to the 20 children. Participants in the unit asking condition were asked to first indicate how much they hypothetically would donate to one child (i.e., one unit), and thereafter they were asked how much they were willing to donate to the 20 children.

### Results

For all experiments reported in this paper, we screened the data for outliers. In line with our pre-registration, we excluded participants who donated values beyond three standard deviations from the mean (like Hsee et al. [27]). This was done for each condition separately, resulting in an exclusion of 13 participants from any further analyses in this study (final sample size = 984 participants).

#### WTD

**Fig 1:**
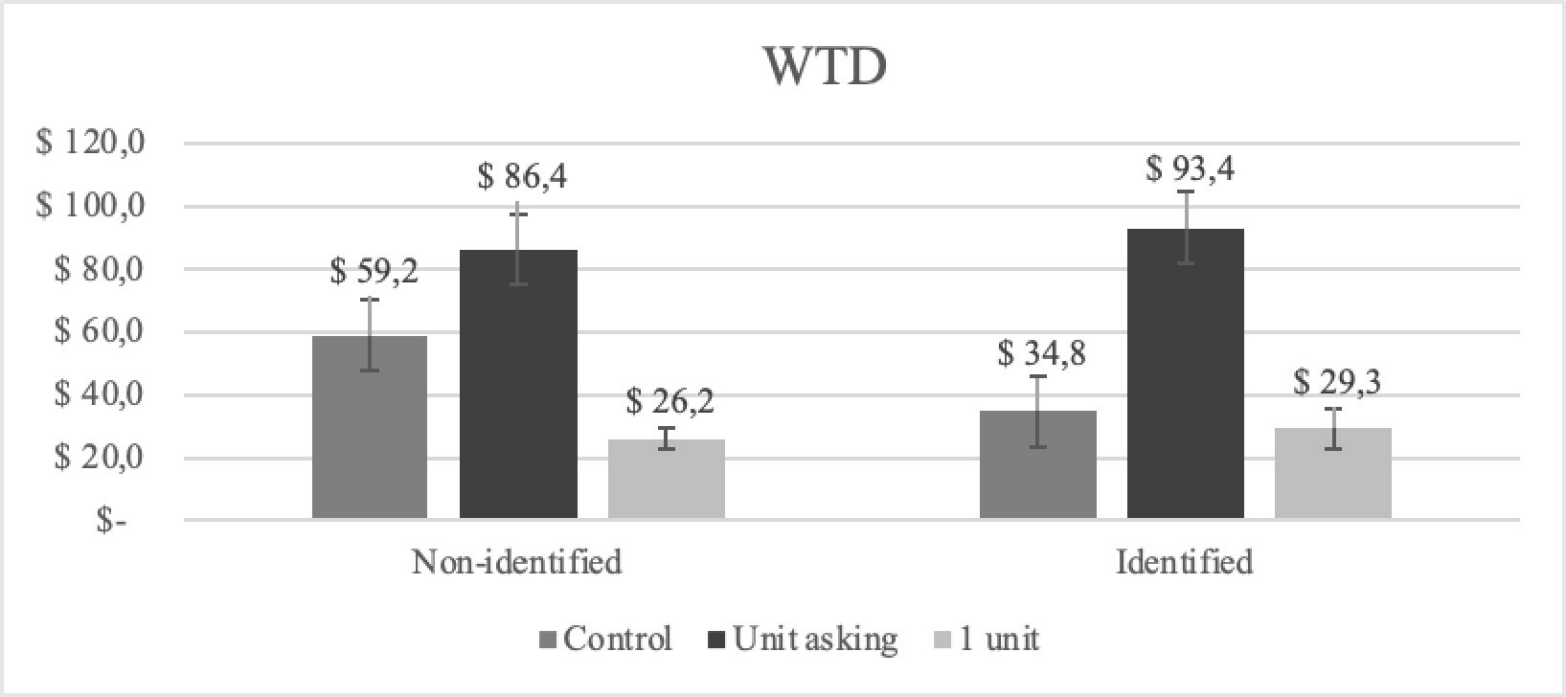
Mean WTD to the 20 children for each condition and to the unit (1 child) in Study 1. Error bars display standard error of mean.

Fig 1 shows the WTD to the 20 children separated for each condition, including the mean WTD to the single unit as indicated by participants in the UA conditions. The results of a 2×2 factorial ANOVA showed that there was a significant UA effect, *F*(1, 980) = 14.84, *p* < .001, *η*_*p*_^*2*^ = .015. People donated significantly more to the 20 children in the UA conditions (*M* = 89.92, *SD* = 224.56) than in the control conditions (*M* = 47.09, *SD* = 103.71). This replicates the UA effect. Further, although people donated less to the 20 children in the identified condition (*M* = 64.20, *SD* = 200.53) than in the non-identified conditions (*M* = 72.64, *SD* = 147.29), there was no significant main effect of identifiability, *F*(1, 980) = 0.61, *p* = .434. Last, there was no significant interaction effect, *F*(1, 980) = 1.99, *p* = .159.

### Discussion Study 1

Study 1 explored how the level of identifiability affects the UA effect in charitable giving. We did this by either including or not including a picture of a single child in the charity appeal. First, we replicated the UA effect [27, 28]. In contrast to our hypothesis, the results indicate that UA was unaffected by IVE (in line with Hsee et al. [27]). But it is important to note that we did not find an IVE in this study. This is similar to other recent studies [16, 34–36], but stands in contrast to the results reported by Hsee et al. [27]. However, it should be noted that the identifiable information in this study was very subtle and possibly a too weak experimental manipulation. Previous studies investigating this effect usually describe the victim with a name and age, and sometimes a short description of the victim’s plight [12, 14]. This was not done here but will be explored in Study 2.

## Study 2

Study 2 extends the design of Study 1 in two major ways: First, we add a third condition that includes more identifiable information. We add this to explore whether more vivid, identifying information will reverse the lack of an identifiability effect that we found in Study 1. This will enable us to investigate whether the IVE is dependent on identifying information that portrays the recipient as an individual (more than a photo; [14]). It also enables us to see how increased affective information in the appeal influences the UA effect (Study 2a). Second, we add affective measures to the study (Study 2b). To investigate whether the identified conditions yield the affective reactions that previous studies have found (e.g., distress and sympathy), we ask another sample to rate the different conditions using affect measures that have been used in previous studies (see below). Study 2 was pre-registered and can be found at https://osf.io/v6947

### Method

#### Participants for Study 2a

A total of 1,229 participants were recruited through Prolific at the end of fall of year 2020. Twenty participants failed the attention check, giving a total sample of 1,209 participants (42.2% women, *M*_*age*_ = 37.75, *SD*_*age*_ = 12.49).

#### Design

The study had a 2(Manipulation group: control vs. unit asking) × 3(Identifiability level: non-identified vs. identified vs. highly identified) between-subject design. Participants were randomly assigned to one of the six conditions. The dependent variable was willingness to donate (WTD) to the 20 children. As in Study 1 and Hsee et al. [27], all donations were hypothetical.

#### Procedure

Participants were presented with the same scenario as in Study 1 (i.e., Christmas gifts to kindergarten). The non-identified and the identified condition were the same as the two conditions in Study 1 (i.e., without or with a picture). In the highly identified condition, participants saw the picture and read added text in the scenario, stating that “*Laura*, *4 years old*, *goes to this kindergarten and comes from a low-income family*. *Her parents are poor and can’t afford buying her gifts*. *Their income is barely enough to pay for rent and food*. *Laura enjoys kindergarten as she can play with toys that she does not have at home*.*”*

Participants in the control condition were asked to indicate their WTD to the 20 children after reading the information. Participants in the UA condition were first asked to indicate their hypothetical WTD to one child (i.e., one unit), before deciding their total amount to donate to the 20 children. Participants answered demographic questions last.

#### Study 2b - Affective reactions rating study

For the affective reaction rating study, we randomized 616 participants from Prolific to one of six conditions in the same 2×3 between-subject design as in Study 2a. However, 20 failed the attention check and were excluded. Thus, our final sample consisted of 596 participants (48.7% women, *M*_*age*_ = 34.09, *SD*_*age*_ = 12.71). Participants saw and read the exact same charity appeal in each condition as participants in the main study did. However, instead of indicating their WTD after reading the scenario, participants answered seven affective measures in a randomized order, aimed to capture their affective reactions to the scenario. The questions were taken from previous studies that have investigated mechanisms for the IVE (or singularity effect) [12, 14, 23].

Taken from Erlandsson et al. [12], personal distress was measured by asking how participants felt when reading the story, using three statements (*“I feel downhearted/sad/emotionally uneasy”*), and further, sympathy was measured by answering another three statements (*“I feel intense compassion/strong empathic feelings/emotionally touched”*) on a 7-point Likert scale (*0 = Do not agree at all*, *6 = Agree completely*). Taken from Kogut and Ritov [14], participants rated their agreement with the statements “*After reading the children’s story I felt worried*, *upset and sad*” (measuring distress) and “*I felt sympathy and compassion towards the poor children*” (measuring empathic concern) on a 7-point Likert scale (*1 = Not at all*, *7 = Very much)*. Taken from Västfjäll et al. [23], participants answered the questions “*How positive do you feel about helping the children*?” and “*How negative do you feel about helping the children*?” on a 6-point Likert-scale (*0 = Not at all*, *5 = Very much*), as well as the question “*How do you feel about donating*?” on a 7-point Likert-scale (*-1 = Slightly negative*, *5 = Positive*). After having answered these measures, participants also indicated their WTD according to the condition they were assigned to. The results of the WTD for the affective reaction study can be found in the S1 Appendix.

### Results

#### Study 2a –WTD

**Fig 2:**
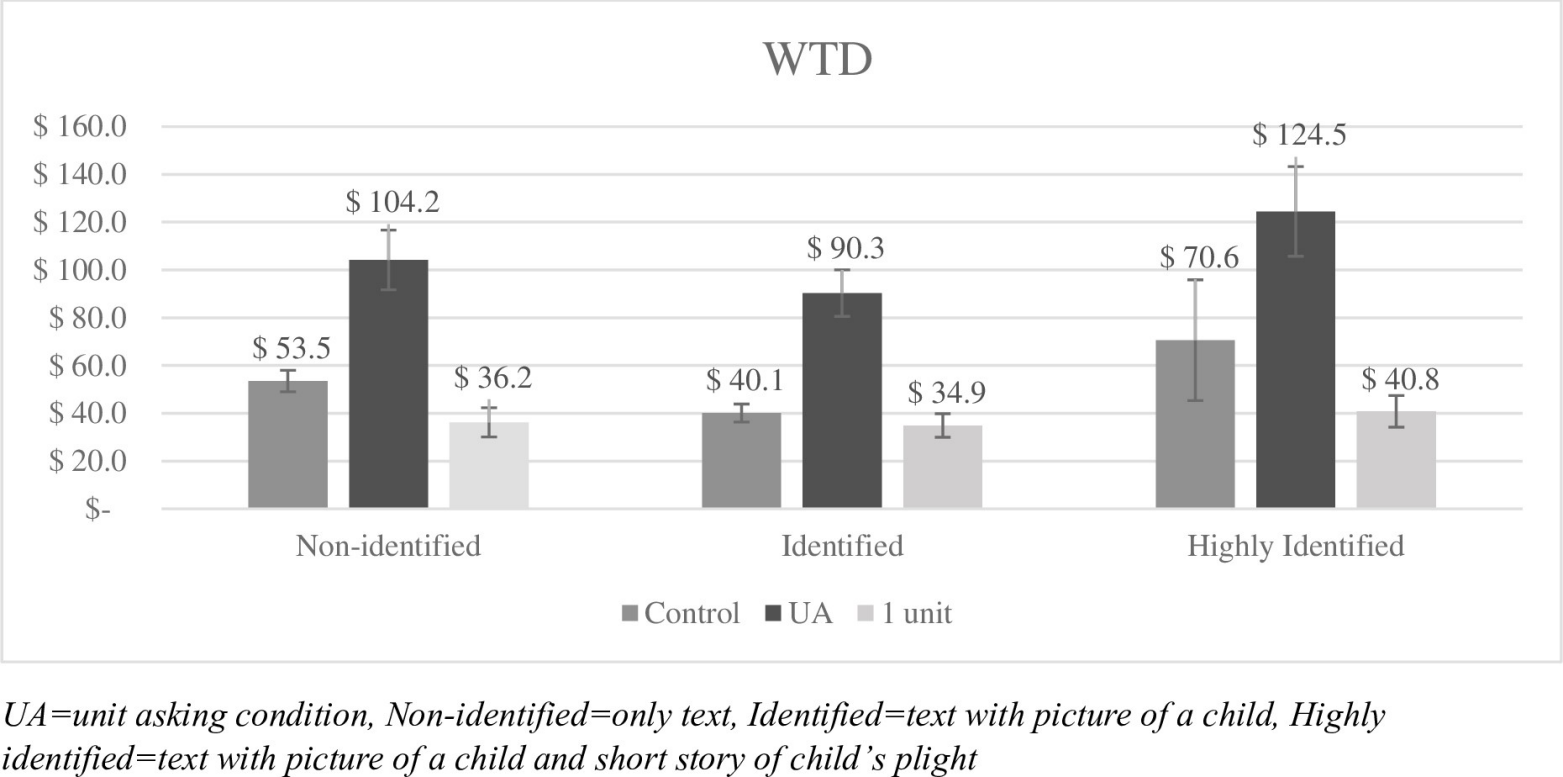
Mean WTD to the 20 children for each condition and to the unit (1 child) in Study 2a. Error bars display standard error of mean. UA = unit asking condition, Non-identified = only text, Identified = text with picture of a child, Highly identified = text with picture of a child and short story of child’s plight.

Fifteen participants who donated beyond three SD’s from the mean were excluded, resulting in a final sample size of 1,194 participants. Fig 2 shows WTD to the 20 children separated by each condition, including the mean WTD to the single unit as indicated by participants in the UA conditions. The results of a 2×3 factorial ANOVA show that there was a significant UA effect, *F*(1, 1188) = 18.62, *p* < .001, *η*_*p*_^*2*^ = .015. People donated significantly more to the 20 children in the UA conditions (*M* = 106.30, *SD* = 200.57) than in the control conditions (*M* = 54.78, *SD* = 212.83). Further, although participants in the highly identified condition donated more to the 20 children (*M* = 97.52, *SD* = 315.63) than participants in the non-identified condition (*M* = 78.97, *SD* = 134.79) and in the identified condition (*M* = 65.43, *SD* = 107.85), we found no significant main effect of identifiability, *F*(2, 1188) = 2.46, *p* = .086. Last, there was no significant interaction effect, *F*(2, 1188) = 0.01, *p* = .991.

#### Study 2b - Affective reaction rating study

A full description of the means and standard deviations for the seven affective measures, divided by the three levels of identifiability and the two levels of manipulation group, can be found in Table 2. Further, a description of the statistical analyses of a 2x3 factorial ANOVA, with identifiability and manipulation group as independent variables and each affective measure as a dependent variable, can be found in Table 3.

**Table 2:** Means (SD) for all seven affective reaction measures for Study 2b, separated by the three levels of identifiability and the two levels of manipulation group.

M *(SD)*	Identifiability			Manipulation group	
	Non-identified	Identified	Highly identified	Control condition	Unit asking condition
Personal distress	2.79 *(1*.*56)*	2.98 *(1*.*53)*	3.53 *(1*.*57)*	3.18 *(1*.*58)*	3.02 *(1*.*59)*
Sympathy	4.02 *(1*.*49)*	3.96 *(1*.*47)*	4.26 *(1*.*47)*	4.13 *(1*.*47)*	4.03 *(1*.*49)*
Distress	3.91 *(1*.*70)*	3.84 *(1*.*72)*	4.50 *(1*.*68)*	4.17 *(1*.*69)*	4.00 *(1*.*76)*
Empathic concern	5.48 *(1*.*42)*	5.42 *(1*.*46)*	5.67 *(1*.*38)*	5.58 *(1*.*35)*	5.46 *(1*.*49)*
Positive feelings	4.12 *(1*.*04)*	3.99 *(1*.*01)*	3.95 *(1*.*27)*	4.01 *(1*.*12)*	4.03 *(1*.*11)*
Negative feelings	0.57 *(1*.*07)*	0.71 *(1*.*14)*	0.70 *(1*.*25)*	0.69 *(1*.*22)*	0.63 *(1*.*09)*
Donation feelings	3.64 *(1*.*44)*	3.50 *(1*.*37)*	3.63 *(1*.*53)*	3.65 *(1*.*45)*	3.53 *(1*.*44)*

**Table 3:** Statistical results for a 2x3 factorial ANOVA with the seven affective reaction measures as the dependent variable and identifiability and manipulation group as the independent variables, for Study 2b.

	Main effect: Identifiability	Main effect: Manipulation group	Interaction effect
Personal distress	*F*(2, 590) = 12.18, *p* < .001, *n*_*p*_^*2*^ = .040	*F*(1, 590) = 1.48, *p* = .224	*F*(2, 590) = 0.17, *p* = .843
Sympathy	*F*(2, 590) = 2.17, *p =* .116	*F*(1, 590) = 0.77, *p* = .381	*F*(2, 590) = 0.11, *p* = .894
Distress	*F*(2, 590) = 9.14, *p* < .001, *n*_*p*_^*2*^ = .030	*F*(1, 590) = 1.44, *p* = .230	*F*(2, 590) = 0.11, *p* = .897
Empathic concern	*F*(2, 590) = 1.66, *p =* .192	*F*(1, 590) = 1.21, *p* = .272	*F*(2, 590) = 0.18, *p* = .835
Positive feelings	*F*(2, 590) = 1.15, *p* = .319	*F*(1, 590) = 0.02, *p* = .883	*F*(2, 590) = 0.42, *p* = .658
Negative feelings	*F*(2, 590) = 0.94, *p* = .392	*F*(1, 590) = 0.49, *p* = .486	*F*(2, 590) = 0.94, *p* = .390
Donation feelings	*F*(2, 590) = 1.03, *p* = .310	*F*(1, 590) = 0.49, *p* = .486	*F*(2, 590) = 1.32, *p* = .267

We found a significant difference between levels of identifiability for two measures, the measure of personal distress (*α* = .88) taken from Erlandsson et al. [12] and the distress item taken from Kogut and Ritov [14] (see Table 2). Bonferroni post-hoc test showed that participants in the highly identified conditions reported significantly higher personal distress and distress compared to those in the non-identified conditions (personal distress: *p* < .001; distress: *p* = .002), and the identified conditions (personal distress: *p* = .001; distress: *p* < .001), but no difference between non-identified and identified conditions (personal distress: *p* = .67; distress: *p* = 1.00). Further, we also correlated these two measures with WTD and found a significant, negative correlation for personal distress in the identified control condition (*r* = -.200, *p* < .05), and a significant, positive correlation in the highly identified, UA condition (*r* = .219, *p* < .05). Further, we found a significant, positive correlation with the distress measure in the highly identified conditions (control condition: *r* = .261, unit asking condition: *r* = .299). The correlation for the distress measure was also negative in the control condition (*r* = -.172) but did not reach significance. The remaining correlations were positive but insignificant (ranging from *r* = .007 to *r* = .195).

### Discussion Study 2

In Study 2, we extend the IVE manipulation as well as test for what kinds of affective reactions the identifying information elicited. As in Study 1, UA boosted WTD. In contrast to our predictions, we did not find that the IVE significantly interacted with UA. However, since we did not find a main effect of the IVE in this study either we cannot conclude that IVE and UA are independent. The results from Study 2b suggest that increased identifiability increases experienced distress, similar to previous research [12, 14, 19].

## Study 3 –IVE, mental imagery, and order effects

Since we did not find an IVE in either of the two previous studies, we cannot properly examine the relationship between the IVE and UA. Therefore, Study 3 was designed to increase the likelihood of finding an IVE. Dickert et al. [37] hypothesized that one of the cognitive and affective mechanisms driving the IVE is mental imagery. They found results indicating that a more clear mental image of the victim was related to higher sympathy for that victim and higher subsequent donations. Building on this idea, we attempted to develop a material that would help participants create a vivid mental image of the donation recipient to enhance the IVE effect. Mental imagery has been suggested to work similarly to our perception [38], and can in a similar way as our perception impact our emotions and subsequent actions [39–42]. Thus, we reasoned that creating enhanced mental imagery of the donation recipient will increase a range of empathic affective reactions towards that recipient and alter subsequent donation behavior.

Study 3 does not include the UA method used in studies 1 and 2, but only attempts to produce a reliable IVE material. We do, however, test whether the ratings of affect before or after stating WTD systematically impact WTD. We reasoned that making the affective reactions to the victim salient to donors before making a donation decision could cause them to cue off these salient feelings more (relative to donors not rating their affect first)–thus leading to higher donation levels.

### Method

#### Participants

A total of 1,503 participants from Prolific completed the study during the beginning of fall 2022. Three participants failed the attention check, and the final sample consisted of 1,500 participants (*M*_age_ = 37.34, *SD* = 13.06, 50.2% female).

#### Design

Participants were randomly assigned to a condition in a 2 (Order: emotions first vs. donations first) x 3 (Identifiability: non-identified vs. identified vs. highly identified) between-subject design. The first factor referred to whether participants, after having read about the charitable cause of the poor children at the kindergarten (identical to Study 2), were asked to first rate their affects, using the same measures as in Study 2b, or first indicate how much to donate. All participants answered both affective reactions and WTD, albeit in a different order. The second factor referred to the level of identifiability in the charitable cause, identical to the three levels from Study 2 (i.e., non-identified, identified, highly identified).

#### Procedure

The procedure was identical to that of Study 2a, with three crucial exceptions; 1) participants did not undergo the UA method in any condition, 2) participants rated both their affect and indicated WTD, but in different orders. When answering the affective measures, participants were asked to rate how they felt when considering the children at the kindergarten. The affective measures were presented in the following order for all participants: personal distress, sympathy, distress, empathic concern, positive feelings, negative feelings, and last, donation feelings. 3) The conditions with some identifying information (either only a picture or a picture and a personalized story) were amplified with a mental imagery task. Participants in these conditions were presented with an image of a donation recipient and were then asked to create “a vivid mental image” scaffolded by questions aimed to increase the identifiability of the recipient by for example positioning the recipient (adapted from Holmes et al. [43], Pile et al. [44]):

*“Now please build up a very vivid mental image of the situation described below*, *using all of your senses*. *Imagine that it is Christmas day and the child do not receive any Christmas gift*, *although she/he really has hoped for one*.

***Example question and answer***

*Where is the child*?

In her/his family home.

*Where is the child and what does she/he look like*? *What is her/his facial expression*? *What does she/he feel*? *Can you hear anything*, *if yes what*?*”*

Participants could describe their mental imagery in their own words in a free text box. Last, before answering demographic questions, all participants also answered how vividly they had imagined a child in their mind’s eyes by choosing one of five options; “No image at all (only “knowing” that you are thinking of a child)”, “Vague and dim”, “Moderately clear and vivid”, “Clear and reasonably vivid”, or “Perfectly clear and as vivid as normal vision”. All participants that underwent the mental imagery task (identified or highly identified conditions) had a more vivid image of a child in their mind than those in the non-identified condition (all *p*’s < .001), suggesting that the identified information along with the mental imagery task increased how clear they imagined a single, identified child.

### Results

We conducted a 2x3 factorial ANOVA with the affective reactions or WTD as the dependent variables and order and identifiability as independent variables. WTDs over 3 SDs over the mean per condition were first winsorized before conducting the analysis. The ANOVA found a significant order effect, *F*(1, 1494) = 8.290, *p* < .001, *n*_*p*_^*2*^ = .006, indicating that participants donated significantly more if they first rated their affective reactions (*M* = 59.53, *SD* = 111.82) than if they first indicated how much they wanted to donate (*M* = 46.18, *SD* = 58.04). There was no significant effect of identifiability, *F*(2, 1494) = 0.480, *p* = .618. We found no IVE also when we exploratory compared the control condition (no identifying information) to both conditions with identifying information combined using an independent t-test, *t*(1498) = -0.835, *p* = .404. Further, there was no interaction effect, *F*(2, 1494) = 0.1430, *p* = .867.

#### Affective reactions

Means and standard deviations for the affective reaction measures for the two independent variables can be seen in Table 4. All statistical results for the seven affective reaction ratings can be seen in Table 5. There was a significant order effect for personal distress and negative feelings, indicating that participants rated they experienced significantly more personal distress and negative feelings if they first rated their affective reactions than if they first indicated how much they wanted to donate. There was also a main effect of identifiability for four of the measures: personal distress, sympathy, distress, and empathic concern. For all measures, participants in the identified condition or in the highly identified condition rated affect as significantly more intense compared to the non-identified condition, whereas there was no significant difference between the two imagery conditions for any of the measures.

**Table 4:** Means (SD) for all seven affective reaction measures for Study 3, separated by the three levels of identifiability and the two levels of order.

M *(SD)*	Identifiability			Order effect	
	Non-identified	Identified	Highly identified	Emotions first	Donations first
Personal distress	3.33 *(1*.*69)*	4.15 *(1*.*57)*	4.09 *(1*.*61)*	3.95 *(1*.*65)*	3.74 *(1*.*68)*
Sympathy	4.13 *(1*.*54)*	4.51 *(1*.*49)*	4.45 *(1*.*47)*	4.38 *(1*.*46)*	4.33 *(1*.*55)*
Distress	3.88 *(1*.*82)*	4.89 *(1*.*73)*	4.64 *(1*.*78)*	4.53 *(1*.*79)*	4.39 *(1*.*86)*
Empathic concern	5.23 *(1*.*55)*	5.59 *(1*.*49)*	5.51 *(1*.*52)*	5.45 *(1*.*50)*	5.43 *(1*.*55)*
Positive feelings	3.89 *(1*.*19)*	3.93 *(1*.*27)*	3.89 *(1*.*27)*	3.94 *(1*.*21)*	3.86 *(1*.*27)*
Negative feelings	0.61 *(1*.*05)*	0.64 *(1*.*17)*	0.65 *(1*.*17)*	0.70 *(1*.*18)*	0.57 *(1*.*07)*
Donation feelings	3.69 *(1*.*36)*	3.78 *(1*.*45)*	3.71 *(1*.*45)*	3.71 *(1*.*40)*	3.74 *(1*.*44)*

**Table 5:** Statistical results for a 2x3 factorial ANOVA with the seven affective reaction measures as the dependent variable and order and identifiability as the independent variables, for Study 3.

	Main effect: Identifiability	Main effect: Order	Interaction
Personal distress	*F*(2, 1494) = 41.176, *p* < .001, *n*_*p*_^*2*^ = .052	*F*(1, 1494) = 5.983, *p* = .015, *n*_*p*_^*2*^ = .004	*F*(2, 1494) = .807, *p* = .446
Sympathy	*F*(2, 1494) = 9.386, *p* < .001, *n*_*p*_^*2*^ = .012	*F*(1, 1494) = 0.427, *p* = .514	*F*(2, 1494) = 0.540, *p* = .583
Distress	*F*(2, 1494) = 44.627, *p* < .001, *n*_*p*_^*2*^ = .056	*F*(1, 1494) = 2.354, *p* = .124	*F*(2, 1494) = .807, *p* = .446
Empathic concern	*F*(2, 1494) = 7.827, *p* < .001, *n*_*p*_^*2*^ = .010	*F*(1, 1494) = 0.074, *p* = .785	*F*(2, 1494) = 0.963, *p* = .382
Positive feelings	*F*(2, 1494) = 0.203, *p* = .816	*F*(1, 1494) = 1.694, *p* = .193	*F*(2, 1494) = 0.937, *p* = .605
Negative feelings	*F*(2, 1494) = 0.227, *p* = .797	*F*(1, 1494) = 4.325, *p* = .038, *n*_*p*_^*2*^ = .003	*F*(2, 1494) = 0.753, *p* = .471
Donation feelings	*F*(2, 1494) = 0.522, *p* = .593	*F*(1, 1494) = 0.168, *p* = .682	*F*(2, 1494) = 0.572, *p* = .563

### Discussion Study 3

Although we aimed to increase the chances of finding an IVE for donations in Study 3, we were not able to achieve this goal even when combining the IVE with a mental imagery manipulation to boost affect. However, we did find an IVE for several of the affective measures, suggesting that our manipulation was successful in inducing the intended affective reactions. For example, participants in one of the more identified conditions (identified or highly identified conditions) experienced significantly more sympathy and distress than those in the non-identified condition.

In addition, we found an order effect, both on donations and on two of the affective reaction measures (personal distress and negative feelings), where participants that first rated their affective reactions donated more and had stronger affective reactions. This finding is in line with research showing that affect intensity is amplified when made salient [45] as well as “context effects” [46]. More broadly, this finding is consistent with the notion of preference construction [47]–that people do not have strong pre-existing values but instead use available information when expressing preferences. Thus, when donors’ affective reactions are made salient by having them rate them first, they use this salient information when answering the question “how much should I donate?”. We tentatively call this an "emotion asking" effect and explore this effect further in Study 4, along with the UA method and a new stimulus material for the IVE.

## Study 4 –IVE, UA, and emotion asking

In Study 4 we shift to another stimulus material that previously resulted in a robust IVE for donations (i.e., Study 4 by Erlandsson et al. [12]). This material thus should be effective in inducing a reliable IVE as well as helps in extending the material beyond that used by Hsee et al. [27]. In addition, we follow up on the “emotion asking” effect found in Study 3 by including this manipulation also here. Study 4 was pre-registered and can be found at https://osf.io/rv94n.

### Method

#### Participants

A total of 1,701 participants ((*M*_*age*_ = 37.32, *SD* = 12.92, 49.9% female) from Prolific completed the study during the mid of fall 2022, but seven of these failed the attention check.

#### Design

Participants were randomly assigned to one condition in a 2(Identifiability: non-identified vs. identified) x 2(Manipulation group: control vs. unit asking) x 2(Order: emotions first vs. donations first) between-subject design. First, participants would either read some general information about a hospital treating and fighting childhood cancer, or read a personal letter written by parents about a child that had died from cancer, described with name, age, a picture, and some personal characteristics. Second, participants would either undergo the UA method, where they first were asked to indicate their WTD to one child and later to all 20 children, or control condition. Last, participants would either first rate their affective reactions towards the children or first indicate WTD.

#### Procedure

After information, consent, and an attention check, participants were randomized to one of eight conditions. To keep most information similar to the scenario with the poor children at the kindergarten (and similar to Study 1 by Hsee et al. [27]), participants were asked to imagine that they received a letter from a doctor at a local hospital they knew personally who was asking for donations. Participants in the unidentified condition would read a description that the hospital contributes as a financier in the treatment and research for childhood cancer. There was some information about their work and how childhood cancer can affect children and families. It also stated that the hospital was currently treating 20 children with cancer who did not have health insurance and was asking for donations to help these sick children. The exact description of the stimuli can be found in the S1 Appendix. Participants in the identified conditions instead read a personal letter written by the parents of a girl who had died of cancer, describing the child and the journey with cancer. The information about the hospital having 20 children was also included as it was needed for the UA method. Further, the order of first indicating WTD or first rating one’s affective reaction towards the children at the hospital was similar to the procedure from Study 3 and the UA method was similar to studies 1–2. An exception was that for those in the UA condition who first rated their affects, they indicated WTD to a single child first, then followed by the affective measures (which asked participants to rate how they felt when considering the children at the hospital) and last, they indicated WTD to the 20 children. The survey ended with demographic questions.

### Results

Due to severely skewed data for WTD measure, even after having removed data points that were over 3 SDs from the mean or after winsorizing, we decided to instead remove all data points that had indicated WTD that was more than $500 per condition. A total of 62 participants (from eight conditions) were removed from further analyses leaving a final sample of 1,632 participants. The main analyses were a factorial ANOVA with identifiability, manipulation group, and order as independent variables and either WTD or one of the affective measures as the dependent variable.

#### WTD

**Fig 3:**
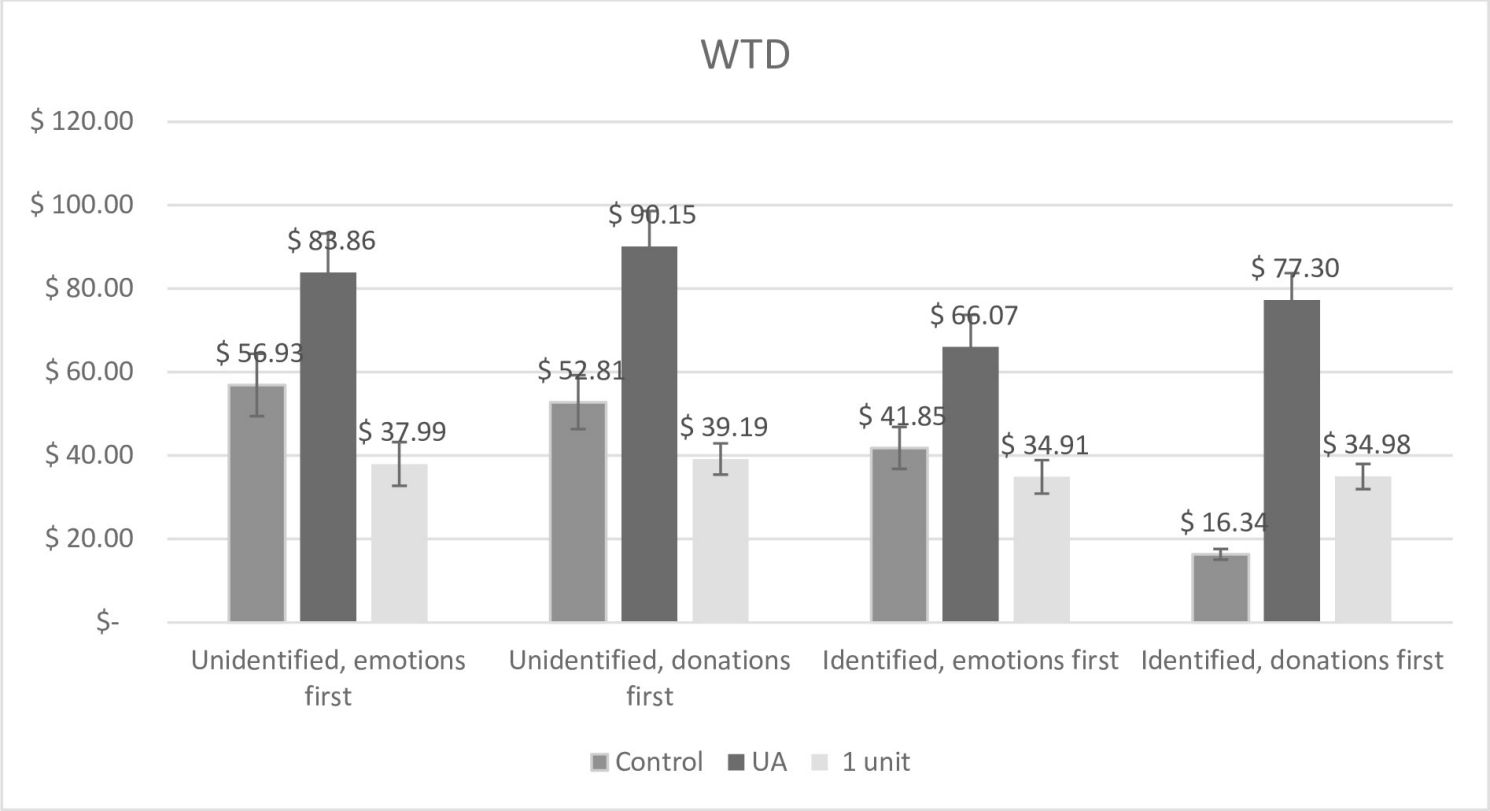
Mean WTD to the 20 children for each condition and to the unit (1 child) in Study 4. Error bars display standard error of mean.

Fig 3 shows the WTD to the 20 children separated by each condition, including the mean WTD to the single unit as indicated by participants in the UA conditions. The results from the ANOVA showed that there was a main effect of identifiability, *F*(1, 1624) = 16.27, *p* < .001, *n*_*p*_^*2*^ = .010. However, this was the opposite of an IVE, since participants seeing an identified appeal donated significantly less (*M* = 55.51, *SD* = 97.52) than participants seeing an unidentified appeal (*M* = 70.70, *SD* = 108.18). Further, we found a UA effect, *F*(1, 1624) = 53.79, *p* < .001, *n*_*p*_^*2*^ = .032, such that participants in the UA condition donated significantly more (*M* = 78.78, *SD* = 117.10) than participants in the control condition (*M* = 41.94, *SD* = 76.77). Last, we also found an interaction effect between manipulation group and emotion asking, *F*(1, 1624) = 5.35, *p* = .021, *n*_*p*_^*2*^ = .003. Participants in the control conditions donated significantly more if they first rated their emotions (*M* = 49.33, *SD* = 85.45) than if they first indicated how much to donate (*M* = 34.77, *SD* = 66.63; *p* = .046), whereas participants in the UA did not donate significantly differently whether they first rated their emotions (*M* = 74.58, *SD* = 115.00) or first indicated how much to donate (*M* = 81.67, *SD* = 118.55; *p* = 210). The two other possible interaction effects were not significant (both *p*´s > .305) and the three-way interaction was not significant either (*p* = .196).

#### Affective reactions

Means and standard deviations for the affective reaction measures for the three independent variables can be seen in Table 6. First, there was a main effect of identifiability for personal distress, *F*(1, 1624) = 13.34, *p* < .001, *n*_*p*_^*2*^ = .008, as well as for distress, *F*(1, 1624) = 9.07, *p* = .003, *n*_*p*_^*2*^ = .006. Participants seeing the identified appeal rated they experienced significantly higher levels of personal distress and distress compared to participants seeing the unidentified appeal. Second, there was no significant main effect of the manipulation group (control vs. unit asking method; all *p*´s > .059) or a significant main effect of order (emotion asking; all *p*´s > .301) for any of the affective measures. Third, there was a recurrent interaction effect between identifiability and manipulation group for three measures: personal distress, *F*(1, 1624) = 4.09, *p* = .043, *n*_*p*_^*2*^ = .003, sympathy, *F*(1, 1624) = 4.75, *p* = .029, *n*_*p*_^*2*^ = .003, and empathic concern, *F*(1, 1624) = 8.80, *p* = .003, *n*_*p*_^*2*^ = .005, and near significance for distress (*p* = .052). In the control condition, participants seeing the identified appeal experiences significantly more personal distress (*M* = 4.28, *SD* = 1.58), more sympathy (*M* = 4.67, *SD* = 1.46), and more empathic concern (*M* = 5.84, *SD* = 1.44) than participants seeing the unidentified appeal (personal distress: *M* = 3.82, *SD* = 1.53, *p* < .001; sympathy: *M* = 4.42, *SD* = 1.45, *p* = .021; empathic concern: *M* = 5.51, *SD* = 1.46, *p* = .003), whereas this difference was not evident in the UA conditions (personal distress: *M* = 4.08, *SD* = 1.61 vs. *M* = 3.96, *SD* = 1.64, *p* = .234; sympathy: *M* = 4.41, *SD* = 1.56 vs. *M* = 4.49, *SD* = 1.48, *p* = .473; empathic concern: *M* = 5.55, *SD* = 1.52 vs. *M* = 5.66, *SD* = 1.34, *p* = .253). Last, there was another interaction effect between manipulation group and order for positive feelings, *F*(1, 1624) = 4.01, *p* = .045, *n*_*p*_^*2*^ = .002. Participants in the control condition that first rated their emotions experienced significantly higher positive feelings (*M* = 3.66, *SD* = 1.28) than participants in the UA condition that first rated their emotions (*M* = 3.44, *SD* = 1.46, *p* = .032), but if participants first indicated how much to donate, positive feelings did not differ between participants in the control condition (*M* = 3.49, *SD* = 1.40) and the UA condition (*M* = 3.53, *SD* = 1.36, *p* = .530). The last possible interaction effect, between identifiability and order, was not significant for any of the affective measures (all *p*´s > .553), and neither was the three-way interaction (all *p*´s > .627).

**Table 6:** Means (SD) for all seven affective reaction measures in Study 4, separated by the two levels of identifiability, manipulation group, and order.

M (*SD)*	Identifiability		Manipulation group		Order	
	Unidentified	Identified	Control	Unit asking	Emotions first	Donations first
Personal distress	3.89 *(1*.*58)*	4.16 *(1*.*60)*	4.05 *(1*.*57)*	4.04 *(1*.*62)*	4.03 *(1*.*56)*	4.05 *(1*.*62)*
Sympathy	4.45 *(1*.*47)*	4.51 *(1*.*52)*	4.55 *(1*.*46)*	4.44 *(1*.*53)*	4.52 *(1*.*42)*	4.46 *(1*.*56)*
Distress	4.80 *(1*.*66)*	5.03 *(1*.*71)*	5.00 *(1*.*68)*	4.87 *(1*.*70)*	4.90 *(1*.*66)*	4.95 *(1*.*72)*
Empathic concern	5.59 *(1*.*40)*	5.67 *(1*.*49)*	5.68 *(1*.*45)*	5.60 *(1*.*45)*	5.64 *(1*.*40)*	5.63 *(1*.*49)*
Positive feelings	3.59 *(1*.*35)*	3.49 *(1*.*40)*	3.58 *(1*.*35)*	3.49 *(1*.*40)*	3.55 *(1*.*38)*	3.51 *(1*.*38)*
Negative feelings	0.86 *(1*.*26)*	0.92 *(1*.*29)*	0.83 *(1*.*23)*	0.95 *(1*.*31)*	0.91 *(1*.*28)*	0.88 *(1*.*27)*
Donation feelings	3.26 *(1*.*62)*	3.17 *(1*.*69)*	3.25 *(1*.*64)*	3.17 *(1*.*67)*	3.17 *(1*.*68)*	3.24 *(1*.*63)*

### Discussion Study 4

In Study 4, we tested another stimulus set to investigate the IVE in relation to the unit asking method along with the “emotion asking” effect found in Study 3. However, even when using a charity appeal focusing on childhood cancer (that previously has proven effective in producing the IVE; [12]), we once again did not find the IVE for donations. Instead, we found a reversed IVE for WTD in that participants seeing the unidentified charity appeal donated *more* than those seeing the identified appeal. However, for affective reactions, there was an IVE for personal distress [12] and distress [14]–similar to the findings from Study 2b and partly Study 3. Thus, affect spurred by identified victims does not always translate to donation behavior.

Second, we once again found support for the UA effect for donations (but not for any of the affective reactions). Thus, even using another stimulus material, the UA method seems to increase donations.

Third, two interaction effects were found in Study 4; the “emotion asking” effect–that participants donate more if they rate their affective reactions before donating–was evident only in the control condition for donations, and not in the UA condition, suggesting that the UA canceled this effect. Similarly, the effect of identifiability on affective reactions (personal distress, sympathy, and empathic concern)–more affect if seeing an identified appeal than an unidentified appeal–was evident only in the control condition, but not in the UA condition, suggesting once again that the UA canceled this effect.

## Study 5—Singularity and compassion fade

The singularity effect is distinct from, but related to the IVE since this effect is mainly found when the victim is a single person. In the singularity effect, a single identified victim is often helped more than a group of identified victims [21, 22]. In Study 5, we investigate WTD if participants first valuate either one or a few identified victims. As a group of victims seems to trigger less affective reactions than a single person, having a few persons to represent the unit can lead to different outcomes: 1) donors would not anchor on the heightened affective reaction that the single victim arouses leading to a lower WTD, and/or 2) donors will be less influenced by affect and thereby more sensitive to scope [29, 32].

We also investigate how these patterns are influenced by group size. We compare the effect of the UA method when the group consists of 20 versus 200 children. In their discussion, Hsee et al. [27] briefly mention an unpublished paper where they manipulated the number of victims in the group (10 vs. 100 children) with the UA method. They reported finding an interaction effect that suggested that people were more sensitive to the number of victims in the UA condition than in the control condition.

### Method

#### Participants

Participants were recruited from Prolific and a total of 2,028 subjects completed the study during mid-December 2020. Twenty-eight participants failed the attention check and our final sample consisted of 2000 participants (51.4% females, *M*_*age*_ = 33.90, *SD*_*age*_ = 11.85).

#### Design

The experiment had a 2 (Manipulation group: control vs. unit asking) × 2 (Unit size: 1 child vs. 5 children) × 2 (Group size: 20 children vs. 200 children) between-subject design. Participants were randomly assigned to one of eight conditions. The dependent variable was willingness to donate (WTD) to the larger group (20 or 200 children).

#### Procedure

The procedure and the scenarios presented were identical to studies 1 and 2, except for two things. First, when participants read the description from the donation website, they either saw a picture of a single child (as in studies 1 and 2) in the charity appeal or five pictures depicting five children. For participants in the UA conditions, those who saw five children were asked to initially indicate WTD to these five children. Second, the description of the poor children at the kindergarten either stated that the kindergarten had 20 children (as in studies 1 and 2) or 200 children. For participants reading that the kindergarten held 200 children, they were asked to indicate WTD to these 200 children.

### Results

#### WTD

**Fig 4:**
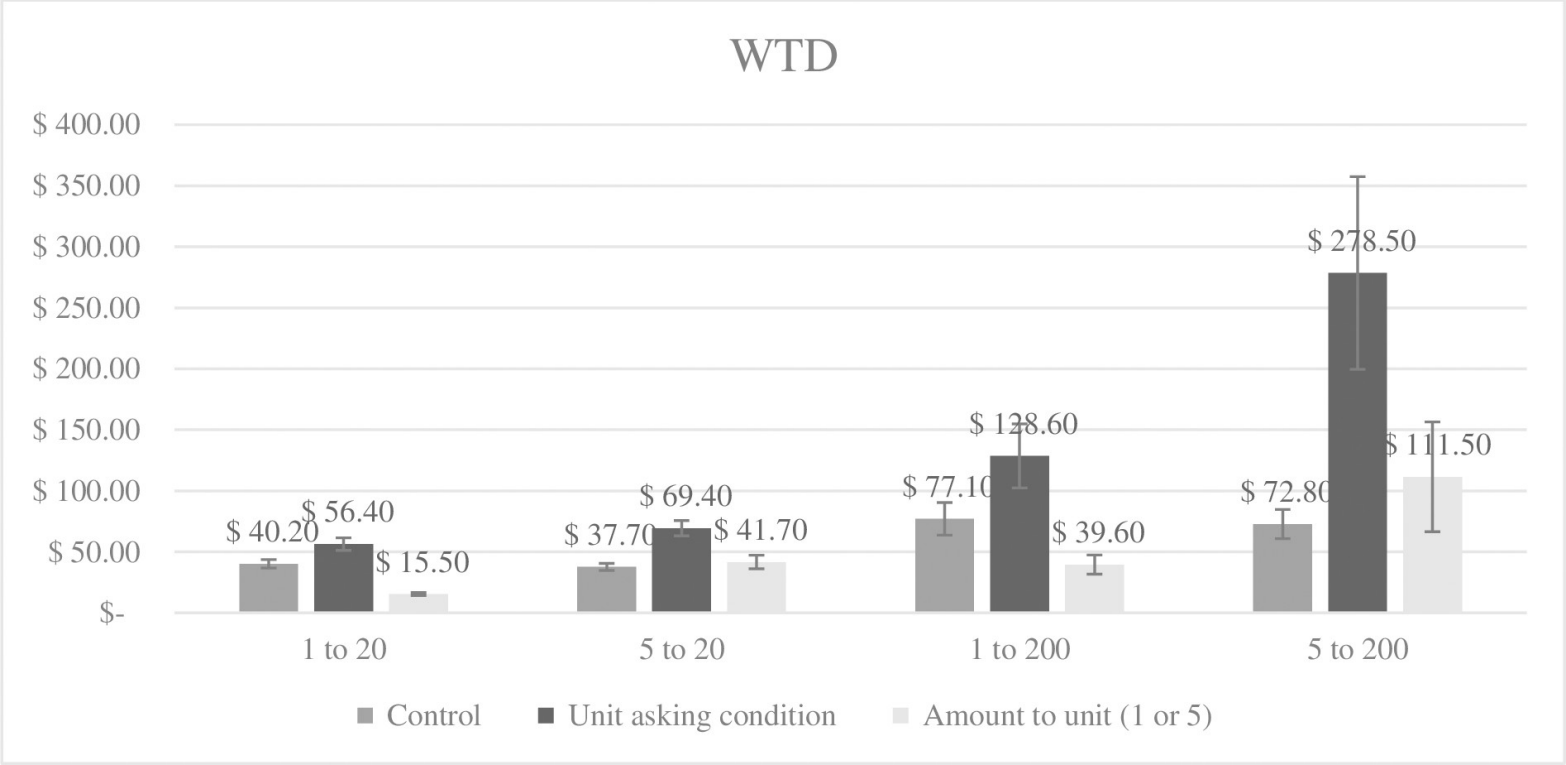
Mean WTD to the big group of children (20 or 200 children) for each condition and to the unit (1 or 5 children) in Study 5. Error bars display standard error of mean.

Twenty-one participants donated higher than 3 SDs over the mean and were excluded. The final sample size was 1,979 participants. Fig 4 shows the mean WTD to the 20 or 200 children separated for all conditions, including the mean WTD to the unit (i.e., one or five children) as stated by participants in the UA conditions.

We conducted a factorial ANOVA with WTD as the dependent variable and intervention group, unit size, and group size as independent variables.

We found two main effects. First, we found a main effect of the intervention, *F*(1, 1971) = 12.55, *p* < .001, *η*_*p*_^*2*^ = .006. Participants in the UA conditions donated significantly more to the whole group of children (*M* = 133.51, *SD* = 665.53) than participants in the control conditions (*M* = 57.07, *SD* = 147.00). Second, there was a main effect of group size, *F*(1, 1971) = 16.84, *p* < .001, *η*_*p*_^*2*^ = .008. Participants donated significantly more if they read that the kindergarten had 200 children (*M* = 139.33, *SD* = 675.78) than if it had 20 children (*M* = 50.97, *SD* = 73.90). There was no main effect of unit size, *F*(1, 1971) = 3.29, *p* = .070. Participants seeing the picture of the single child did not donate significantly more (*M* = 75.71, *SD* = 239.53) than participants seeing the pictures of the five children (*M* = 114.96, *SD* = 640.14).

More importantly, there were two significant interaction effects. First, there was an interaction effect between intervention group and unit size, *F*(1, 1971) = 3.88, *p* = .049, *η*_*p*_^*2*^ = .002. Participants in control conditions did not donate significantly different to the whole group of children whether they saw the picture of one child (*M* = 58.81, *SD* = 155.54) or of five children (*M* = 55.33, *SD* = 138.08; *p* = .911), whereas participants in UA conditions donated significantly more to the whole group of children if they saw five children (*M* = 174.36, *SD* = 890.21) than one child (*M* = 92.58, *SD* = 300.06; *p* = .007). Second, there was an interaction effect between intervention and group size, *F*(1, 1971) = 5.90, *p* = .015, *η*_*p*_^*2*^ = .003. Participants in the control condition did not donate significantly more to a kindergarten with 200 poor children (*M* = 74.98, *SD* = 199.65) than one with 20 poor children (*M* = 38.94, *SD* = 50.26; *p* = .237), whereas participants in the UA conditions did (20 children: *M* = 62.92, *SD* = 90.02 versus 200 children: *M* = 203.67, *SD* = 930.65; *p* < .001). This latter interaction effect is in line with Hsee et al. [27] unpublished study. The last possible interaction effect with unit size and group size was not significant (*p* = .117) and neither was the three-way interaction effect (*p* = .107).

### Discussion Study 5

In Study 5, we investigated how UA related to singularity manipulations (seeing the picture of one or five children) and compassion fade (20 versus 200 poor children). The results show that UA diminished a potential singularity effect, in the sense that participants who saw and valuated five children first (compared to one child) expressed a higher WTD to the group. Similarly, participants in the UA condition expressed a significantly higher WTD to 200 than to 20 children. However, participants in the control condition were rather insensitive to the number of children in their WTD—they did not donate more if seeing five instead of one child, or if the kindergarten had 200 instead of 20 children.

We found a general insensitivity to scope in the control conditions. However, as participants did not express a significantly higher WTD if they saw the picture of one child compared to five children or expressed a significantly lower WTD if there were 200 children in the kindergarten compared to 20 children, we do not find robust evidence for a singularity effect or compassion fade effect. Nevertheless, participants showed a clear insensitivity to quantity [48], as they did not give more if they saw five children, or if the kindergarten had 200 children. However, in the context of UA, the manipulations of increasing unit size and group size were positive for WTD. If there were pictures of five children (instead of one) and if the kindergarten held 200 children (instead of 20), donations increased in the UA effect. Looking at Fig 4, we find something approaching a linear function where donations to the whole group of children increased as unit size increased from one to five children and group size increased from 20 to 200 children.

## General discussion

We set out to systematically combine the identifiable victim effect (IVE), the singularity effect, and unit asking (UA) in a charitable giving context. Given that the IVE and singularity has previously been shown to be driven by affect, and that the UA involves elements of both these effects, this research allowed us to better understand how affect motivates willingness-to-donate (WTD) for single and groups of identified/unidentified victims as well as to what extent affect is a contributing factor in UA.

First, in the studies on the IVE, the results show that UA increased WTD regardless of the level of identifiability. At the surface, this finding suggests that the IVE and UA are unrelated, and that affect is not a strong motivator for WTD. However, in studies 1, 2, and 4 and for Study 3, which was an additional study specially designed to increase the effectiveness of the manipulation, we surprisingly failed to find a main effect of IVE on donations. We did find that the IVE manipulations overall are effective in inducing the intended affective reactions, suggesting that the IVE is a robust affective phenomenon. But since neither of the IVE manipulations nor materials reliably influenced donations in the expected direction, we cannot fully conclude that UA does not depend on the level of identifiability. It is important to note that our IVE manipulations relied on materials that have found effects on donations in previous research [12] and that we used incidental affect-amplifying experimental such as mental imagery, but still failed to find that the stronger affective reactions associated with higher levels of identifiability translated to donations. While the IVE is seen as a robust effect, recent research has repeatedly found null- or negative results using IVE manipulations [34, 35]. Thus, in line with other studies failing to find the effect, our studies suggest that the IVE is not a strong effect that will occur under all conditions. Despite this, there are real-life examples of IVE [25] so its boundary conditions and generalizability across contexts and sample is in dire need of further investigation [49]. The failure to find an IVE effect in the current studies is likely not due to the materials as we used stimuli that previously has been documented to produce IVE effects [12]. It may instead be related to sample characteristics and motivations of participants (i.e. earning money in a study). It may also relate to another important limitation of the current study–the reliance on hypothetical donations. We opted to closely follow Hsee et al.’s [27] study 1 design that used hypothetical donations and an “unbounded” WTD measure. Our results point to that the unbounded nature of the WTD measure is problematic as some participants indicate very high donation amounts. Though it is not uncommon to use hypothetical donations in studies of charitable giving [13–17, 20–24], we acknowledge that this is an important limitation and that hypothetical donations may differ in important ways from real donations [50–52]. However, in the original UA paper Hsee et al. [27] extended their paradigm to real donations in a field experiment and found, again, that an UA effect was evident. Thus, how incentivized and non-incentivized versions of the UA method may differ both in its effect on behavior and the mechanisms underlying it is an open question that future research should examine in different settings (both laboratory and field) and in diverse populations.

In studies 3 and 4, we find a novel “emotion asking” effect–an order effect both on donations and on the affective measures, where participants that first rated their affective reactions donated more and had stronger affective reactions (a form of additive effect along the lines with our first claim). This order effect was evident for both identified and non-identified experimental conditions (Study 3), but in Study 4 which included UA, the emotion asking effect was only evident in the control condition, and not in the UA conditions, suggesting that the UA attenuates this effect (consistent with our second claim). A possible explanation for this comes from Ritov and Baron [29] who showed that joint evaluation (like UA) attenuates the impact of affect on judgments and decisions.

In the study on the singularity effect and compassion fade (Study 5), UA increased sensitivity to numerical information. When participants anchored their first valuation on five children (instead of one) and if the kindergarten had 200 children (instead of 20), donations increased. This was not the case in the control conditions.

Overall, our results indicate that the UA appears to be robust and a replicable way to increase donations [27, 28, 53]. Even when we vary levels of identifying information, vary how big the group is, or change the number of children to anchor on, UA boosted donations. It is however unclear from the present results to what extent UA involves affect as a driving mechanism. Thus, our studies perhaps are better seen as a large-scale investigation of three interrelated effects in charitable giving, rather than a definitive answer to what is the main psychological driver behind UA.

In summary, we think this research contributes in several ways; 1) we simultaneously study IVE, singularity, and UA in an attempt to establish the relative contribution and interplay of these three different, but interrelated, effects in charitable giving, 2) we find that the IVE is a robust effect when it comes to inducing higher levels of affect, but not donations. Thus, our results suggest that UA operates independently of the IVE (and likely independent of affect) and, moreover, that IVE while increasing affective reactions may not increase donations, 3) in four studies we find strong support that UA boosts donations, both when using the original materials from Hsee et al. [27] and new materials, and 3) combining UA with varying numbers of children in need–both to anchor on in the first valuation and in relation to the number of children in the bigger group–effectively make people more sensitive to numbers when making charitable donations.

The results from the five experimental studies presented here have some implications for real-world giving. Many charitable organizations use identified victims and affective narratives to engage donors [25]. This may indeed be helpful to motivate action, but in order maximize giving, affective responses should be combined with deliberation about impact [54]. Unit asking thus appears to be a robust way to increase sensitivity to the scope of suffering while at the same time allowing for donors to react affectively to the charity appeal [25, 53, 55]. Charity organizations could use these techniques to maximize both the donor’s warm glow and the impact for the benefactors.

## Supporting information

S1 Appendix (DOCX)
