# Supplementary material for: Victim identifiability, number of victims, and unit asking in charitable giving
Source: PLoS One. 2024 Mar 28;19(3):e0300863. doi: 10.1371/journal.pone.0300863 (PMC10977801; doi:10.1371/journal.pone.0300863)
Supplement: S1 Appendix — (DOCX) [file pone.0300863.s001.docx]

**S1. Appendix**

**Supplementary materials**

**Affect and charitable giving: The role of victim identifiability, number of victims, and unit asking method**

Content

[**Study 1** 2](#_Toc136436237)

[*Exploratory analyses* 2](#_Toc136436238)

[*Unit asking pattern (increase/decrease/no change)* 2](#_Toc136436239)

[**Study 2a** 4](#_Toc136436240)

[**Results** 4](#_Toc136436241)

[*Exploratory analyses* 4](#_Toc136436242)

[*Unit asking pattern (increase/decrease/no change)* 4](#_Toc136436243)

[**Study 2b** 6](#_Toc136436244)

[Personal distress (taken from Erlandsson et al., 2015) 6](#_Toc136436245)

[Sympathy (taken from Erlandsson et al., 2015) 7](#_Toc136436246)

[Distress (taken from Kogut & Ritov, 2005) 8](#_Toc136436247)

[Empathic concern (taken from Kogut & Ritov, 2005) 9](#_Toc136436248)

[Positive and negative feelings, and donation feelings (taken from Västfjäll et al., 2014) 10](#_Toc136436249)

[Donation data Study 2b 12](#_Toc136436250)

[*Exploratory analyses* 13](#_Toc136436251)

[*Unit asking pattern (increase/decrease/no change)* 14](#_Toc136436252)

[**Study 4** 15](#_Toc136436253)

[*Unidentified stimulus material* 15](#_Toc136436254)

[*Identified stimulus material* 16](#_Toc136436255)

# **Study 1** **Results**

### *Exploratory analyses*

Similar to Hsee et al. (2013), we conducted additional analyses to investigate if participants were sensitive to numbers (hereafter referred to as scope-consistency) to start with. First, for both unit asking conditions, we compared the WTD to the unit with the WTD to the group, using paired-samples t-test. The results show that for the non-identified condition, scope-consistency was exhibited, *t*(242) = -7.02, *p* < .001. Participants donated more to the to the group of 20 children (*M* = 86.4, *SD* = 154.7) than to the single unit (*M* = 26.2, *SD* = 51.9). For the identified condition, scope-consistency was also exhibited, *t*(246) = -4.74, *p* < .001. Again, participants donated more to the to the group of 20 children (*M* = 93.4, *SD* = 276.9) than to the single unit (*M* = 29.3, *SD* = 101.9). Thus, participants in the unit asking conditions were consistent in valuing a group of children as higher than a single child (i.e., scope-consistency).

Further, we compared the WTD to the unit in the unit asking condition with the WTD to the group in the control condition, using an independent t-test. The results show that for the non-identified conditions, participants in the control group donated significantly more to the group of 20 children (*M* = 53.4, *SD* = 104.2) than participants in the unit asking condition donated to the unit (*M* = 26.2, *SD* = 51.9), *t*(489) = 3.65, *p* < .001. Similarly, for the identified conditions, participants in the control condition donated significantly more to the group of 20 children (*M* = 34.8, *SD* = 43.6) than participants in unit asking condition donated to the unit (*M* = 22.7, *SD* = 39.0), *t*(487) = 3.24, *p* = .001. Thus, participants in the control group were sensitive to number of needy children relative participants donating to a single unit.

### *Unit asking pattern (increase/decrease/no change)*

Further, we looked at the pattern of participants who increased, decreased or made no change in their valuation from 1 unit to the 20 children in the unit asking conditions. We compared this across the two levels of identifiability.

Results below exclude participants beyond +3SD for unit asking pattern. There was no significant difference in donation pattern between the two unit asking conditions, $\chi^{2}$(2, N = 490) = .13, *p* = .94. See Table below.

| *Percentage of participants donating more/same/less to 20 children than to 1 unit* | Increase | No change | Decrease |
| --- | --- | --- | --- |
| UA low identifiability (*N* = 243) | 66.3% (*N* = 161) | 31.3% (*N* = 76) | 2.5% (*N* = 6) |
| UA high identifiability (*N* = 247) | 64.8% (*N* = 160) | 32.8% (*N* = 81) | 2.4% (*N* = 6) |

# **Study 2a**

## **Results**

### *Exploratory analyses*

As in study 1 and Hsee et al. (2013), we conducted additional analyses to investigate if scope-consistency was evident to start with. First, we compared the WTD to the unit with the WTD to the group for all three unit asking conditions, using paired-samples t-test. For the non-identified condition, scope-consistency was exhibited, *t*(198) = -6.49, *p* < .001. Participants donated more to the to the group of 20 children (*M* = 104.2, *SD* = 176.2) than to the single unit (*M* = 36.3, *SD* = 86.2). For the identified condition, scope-consistency was also exhibited, *t*(200) = -7.08, *p* < .001. Again, participants donated more to the to the group of 20 children (*M* = 90.3, *SD* = 138.2) than to the single unit (*M* = 34.9, *SD* = 70.0). Last, for the highly identified condition, scope-consistency was also exhibited, *t*(199) = -5.10, *p* < .001. Again, participants donated more to the to the group of 20 children (*M* = 124.5, *SD* = 265.4) than to the single unit (*M* = 40.8, *SD* = 93.5). Thus, participants in the unit asking conditions were consistent in valuing a group of children as higher than a single child (i.e., scope-consistency).

Further, we compared the WTD to the unit in the unit asking condition with the WTD to the group in the control condition, using an independent t-test. For the non-identified conditions, participants in the control group donated significantly more to the group of 20 children (*M* = 53.5, *SD* = 63.0) than participants in the unit asking condition donated to the unit (*M* = 36.2, *SD* = 86.2), *t*(394) = 2.27, *p* = .024, *d* = .228. However, for the identified conditions, participants in the control condition did not donate significantly more to the group of 20 children (*M* = 40.1, *SD* = 52.9) than participants in unit asking condition donated to the unit (*M* = 34.9, *SD* = 70.0), *t*(396) = 0.824, *p* = .410. Similarly, for the highly identified conditions, participants in the control condition did not donate significantly more to the group of 20 children (*M* = 70.6, *SD* = 357.6) than participants in unit asking condition donated to the unit (*M* = 40.8, *SD* = 93.5), *t*(398) = 1.139, *p* = .256. Thus, only in the non-identified conditions were participants in control group sensitive to number of needy children relative participants donating to a single unit.

### *Unit asking pattern (increase/decrease/no change)*

Further, we looked at the pattern of participants who increased, decreased or made no change in their valuation from 1 unit to the 20 children in the unit asking conditions. We compared this across the three levels of identifiability.

Results below exclude participants beyond +3SD for unit asking pattern. There was no significant difference in donation pattern between the two unit asking conditions, $\chi^{2}$(4, *N* = 600) = 2.53, *p* = .637$.$See Table below.

| *Percentage of participants donating more/same/less to 20 children than to 1 unit* | Increase | No change | Decrease |
| --- | --- | --- | --- |
| UA low identifiability (*N* = 199) | 67.3% (*N* = 134) | 29.6% (*N* = 59) | 3.0% (*N* = 6) |
| UA medium identifiability (*N* = 201) | 63.7% (*N* = 128) | 34.8% (*N* = 70) | 1.5% (*N* = 3) |
| UA high identifiability (*N* = 200) | 67.0% (*N* = 134) | 30.0% (*N* = 60) | 3.0% (*N* = 6) |

# **Study 2b**

## Personal distress (taken from Erlandsson et al., 2015)

Items that participants answered: *I feel downhearted, I feel sad, I feel emotionally uneasy*

Likert-scale from 0 *(= Do not agree at all)* to 6 (=*Agree completely)*

Cronbach’s alfa for the three items of personal distress was *a* = .881.

|  | **Personal distress (Erlandsson et al., 2015)** | | |  |
| --- | --- | --- | --- | --- |
|  | Downhearted M (*SD)* | Sad M (*SD)* | Emotionally uneasy M (*SD)* | ***Total*** |
| Control, Non-identified | 2.95 *(1.64)* | 3.17 (*1.73)* | 2.59 (*1.79)* | 2.90 *(1.55)* |
| UA, Non-identified | 2.65 (*1.74)* | 2.97 *(1.79)* | 2.40 (*1.73)* | 2.67 *(1.57)* |
| Control, Identified | 3.11 (*1.70)* | 3.15 *(1.70)* | 2.76 *(1.74)* | 3.01 *(1.52)* |
| UA, Identified | 2.87 *(1.66)* | 3.34 *(1.68)* | 2.65 *(1.76)* | 2.95 *(1.55)* |
| Control, Highly identified | 3.70 *(1.74)* | 3.89 *(1.74)* | 3.27 *(1.82)* | 3.62 (*1.58)* |
| UA, Highly identified | 3.49 *(1.70)* | 3.75 *(1.68)* | 3.08 *(1.90)* | 3.44 *(1.56)* |

## Sympathy (taken from Erlandsson et al., 2015)

Items that participants answered: *I feel intense compassion, I feel strong empathic feelings, I feel emotionally touched*

Likert-scale from 0 *(= Do not agree at all)* to 6 (=*Agree completely)*

Cronbach’s alfa for the three items of personal distress was *a* = .940.

|  | **Sympathy (Erlandsson et al., 2015)** | | |  |
| --- | --- | --- | --- | --- |
|  | Intense compassion M (*SD)* | Empathic feelings M (*SD)* | Emotionally touched M (*SD)* | ***Total*** |
| Control, Non-identified | 3.97 (*1.62)* | 4.13 (*1.51)* | 4.02 (*1.53)* | 4.04 *(1.49)* |
| UA, Non-identified | *3.97* (1.63) | 4.19 (*1.57)* | 3.86 (*1.63)* | 4.01 *(1.51)* |
| Control, Identified | 3.95 *(1.60)* | 4.19 *(1.54)* | 4.01 *(1.70)* | 4.05 *(1.50)* |
| UA, Identified | 3.84 *(1.52)* | 3.93 *(1.53)* | 3.86 *(1.48)* | 3.88 *(1.43)* |
| Control, Highly identified | 4.08 *(1.57)* | 4.56 *(1.40)* | 4.29 *(1.49)* | 4.31 *(1.41)* |
| UA, Highly identified | 4.03 (*1.64)* | 4.36 *(1.56)* | 4.21 *(1.62)* | 4.20 *(1.53)* |

## Distress (taken from Kogut & Ritov, 2005)

Item that participants answered: *After reading the children's story I felt worried, upset and sad*

Likert-scale from 1 *(= Not at all)* to 7 *(= Very much)*

|  | **Distress** M *(SD)* |
| --- | --- |
| Control, Non-identified | 3.96 *(1.71)* |
| UA, Non-identified | 3.87 *(1.69)* |
| Control, Identified | 3.92 *(1.69)* |
| UA, Identified | 3.76 *(1.76)* |
| Control, Highly identified | 4.63 *(1.58)* |
| UA, Highly identified | - 1. (*1.78)* |

## Empathic concern (taken from Kogut & Ritov, 2005)

Item that participants answered: *I felt sympathy and compassion towards the poor children*

Likert-sscale from 1 *(= Not at all)* to 7 *(= Very much)*

|  | **Empathic concern** M *(SD)* |
| --- | --- |
| Control, Non-identified | 5.56 *(1.46)* |
| UA, Non-identified | 5.39 *(1.38)* |
| Control, Identified | 5.51 *(1.38)* |
| UA, Identified | 5.32 *(1.54)* |
| Control, Highly identified | 5.68 *(1.21)* |
| UA, Highly identified | 5.65 (*1.53)* |

## Positive and negative feelings, and donation feelings (taken from Västfjäll et al., 2014)

Items that participants answered regarding feelings: *How positive/negative do you feel about helping the children?*

Likert-scale from 0 *(= Not at all)* to 5 *(= Very much)*

Item that participants answered regarding feelings about donating: *How do you feel about donating?*

Likert-scale from -1 *(= Slightly negative)* to 5 *(= Positive)*

|  | **Västfjäll et al. (2014)** | | |
| --- | --- | --- | --- |
|  | **Positive feelings** | **Negative feelings** | **Donation feelings** |
| Control, Non-identified | 4.05 *(1.11)* | 0.52 *(1.05)* | 3.57 *(1.57)* |
| UA, Non-identified | 4.18 *(0.96)* | 0.62 *(1.10)* | 3.72 *(1.30)* |
| Control, Identified | 4.02 *(1.02)* | 0.82 *(1.29)* | 3.65 *(1.34)* |
| UA, Identified | 3.96 *(1.01)* | 0.60 *(0.96)* | 3.34 *(1.38)* |
| Control, Highly identified | 3.97 *(1.23)* | 0.74 *(1.30)* | 3.73 *(1.45)* |
| UA, Highly identified | 3.94 (*1.32)* | 0.67 (*1.21)* | 3.53 (*1.61)* |

## Donation data Study 2b

After exclusions of WTD above 3SD from the mean, the final sample consisted of 581 participants (95-99 participants per condition). The WTD were indicated after participants had answered all affective measures.

The figure below shows the mean WTD per condition, for control and unit asking conditions, as well as the WTD to a single unit in the unit asking conditions. Error bars show standard error of mean. Here, it could be noted that the pattern of the donations differs from the pattern found in study 2a, Figure 2. One possibility could be that it relates to participants first assessing on their emotions before indicating their WTD. Previous work has found that making one’s affective state more salient has a bigger effect on one’s assessment than if one does not make the affective state salient (Siemer & Reisenzein, 1998).

We conducted a factorial ANOVA with the independent factors of level of identifiability and manipulation group, and WTD to 20 children as dependent variable. We found that participants in the unit asking conditions donated significantly more to the 20 children (*M* = 72.33, *SD* = 112.71) than those in control conditions (*M* = 43.22, *SD* = 125.88), *F*(1, 575) = 8.798, *p* = .003, *η_p_^2^* = .015, meaning we found a unit asking effect. Further, we found no main effect of identifiability, *F*(2, 575) = 1.245, *p* = .289, meaning there was no difference in WTD between different levels of identifiability. Last, there was no interaction effect, *F*(2, 575) = 1.006, *p* = .345.

### *Exploratory analyses*

We also tested to see if participants in the unit asking conditions were consistent in increasing their WTD from 1 unit to 20 children, for the affective reaction rating sample. We also tested to see if there was a significant difference between the WTD in control condition (to 20 children) and the WTD to the unit in the unit asking conditions.

*Comparing Unit WTD vs. Total WTD*

Condition 2: Unit asking, Non-identified

*Test:* Paired-samples t-test: Comparing unit WTD & total WTD (20 children) in non-identified condition

There was a significant increase in WTD from 1 unit to the whole group of 20 children, *t*(95) = -5.06, *p* < .001, meaning scope-consistency was exhibited.

Condition 4: Unit asking, Identified

*Test:* Paired-samples t-test: Comparing unit WTD & total WTD in identified condition

There was a significant increase in WTD from 1 unit to the whole group of 20 children, *t*(96) = -5.320, *p* < .001, meaning scope-consistency was exhibited.

Condition 6: Unit asking, Highly identified

*Test:* Paired-samples t-test: Comparing unit WTD & total WTD in highly identified condition

There was a significant increase in WTD from 1 unit to the whole group of 20 children, *t*(96) = -6.257, *p* < .001, meaning scope-sensitivity was exhibited.

*Comparing Unit WTD vs. Control WTD*

Non-identified conditions

*Test:* Independent t-test: Comparing unit WTD & control WTD for non-identified conditions

There was a significant higher WTD for 20 children in control condition compared to WTD to one child in UA condition (when there no identifiable information), *t*(190) = 4.677, *p* < .001.

Identified conditions

*Test:* Independent t-test: Comparing unit WTD & control WTD for identified conditions

There was a significant higher WTD for 20 children in control condition compared to WTD to one child in UA condition (when there was a picture in the appeal), *t*(194) = 2.203, *p* = .029.

Highly identified conditions

*Test:* Independent t-test: Comparing unit WTD & control WTD for highly identified conditions

There was a significant higher WTD for 20 children in control condition compared to WTD to one child in UA condition (when there was a picture and a personalized story), *t*(190) = 2.872, *p* = .005.

### *Unit asking pattern (increase/decrease/no change)*

Further, we looked at the pattern of participants who increased, decreased or made no change in their valuation from 1 unit to the 20 children in the unit asking conditions. We compared this across the three levels of identifiability.

Results below exclude participants beyond +3SD for unit asking pattern. There was no significant difference in donation pattern between the three unit asking conditions, $\chi^{2}$(4, N = 290) = 3.03, *p* = .553. See Table below.

| *Percentage of participants donating more/same/less to 20 children than to 1 unit* | Increase | No change | Decrease |
| --- | --- | --- | --- |
| UA non-identified (*N* = 96) | 70.8% (*N* = 75) | 20.8% (*N* = 20) | 1.0% (*N* = 1) |
| UA identified (*N* = 97) | 74.2% (*N* = 72) | 22.7% (*N* = 22) | 3.1% (*N* = 3) |
| UA highly identified (*N* = 97) | 69.1% (*N* = 67) | 26.8% (*N* = 26) | 4.1% (*N* = 4) |

# **Study 4**

Below are the stimuli used for the unidentified conditions and the identified conditions in study 4 (adapted from study 4 by Erlandsson et al., 2015).

## *Unidentified stimulus material*

Imagine the following: A doctor from a local hospital sent you an email asking for donations. You know the doctor personally and trust her words. The email directs you to a charity website. Please answer the questions as if you were making actual donation decisions.

DONATION WEBSITE

Thanks for visiting our website. Please read the following carefully and answer the ensuing questions. Even if you are not willing to make a donation, please still answer the questions; you may simply enter $0. You can revise your answers, and your answers will not be recorded until you move on to the next page.

Our hospital is contributing as a financier in the treatment and research for children with cancer. Through hundreds of research projects each year, we try to find new and better methods that can alleviate and cure childhood cancer. Our hope is to be able to solve the riddle of childhood cancer. 

We exist to mitigate uncertainty and grief of the families who suffer here and now. We also advocate cancer-ill children's issues in the community and visit schools and other forums to talk about childhood cancer and what we are doing to combat it. 

Childhood cancer is the main cause of death in children between 1 and 14 years. And every year, families are victims of the unthinkable; a child receives a diagnosis of cancer. But there is hope.

Currently, our hospital treats 20 children with cancer that do not have health insurance. We hope you can donate, so we can use the money to treath these children and learn to help treat other children. 

We want to give a heartily THANK YOU to everyone contributing to us so that we can continue our fight and research against child cancer. 

Together we are strong; together we can make a difference.

## *Identified stimulus material*

Imagine the following: A doctor from a local hospital sent you an email asking for donations. You know the doctor personally and trust her words. The email directs you to a charity website. Please answer the questions as if you were making actual donation decisions.

DONATION WEBSITE

Thanks for visiting our website. Please read the following carefully and answer the ensuing questions. Even if you are not willing to make a donation, please still answer the questions; you may simply enter $0. You can revise your answers, and your answers will not be recorded until you move on to the next page.


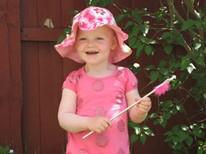

Good night, Mommy 
Good night, Daddy 
The words we would hear every night after the bedtime story ended. 
The words we will never hear you say again.
Amanda, our wonderful girl, almost three years old who loved life and everything it had to offer. 
You loved to sing, dance, swim, and play. You loved to ride merry-go-rounds and have fun at the kindergarten - and you told everyone about it. Every time we passed the amusement park you said: "Look there it is, and it IS open". The warmth and joy you spread to us when riding the carousels was irresistible. 
The hope never left us, but like sand that falls between fingers; your life fell away from us. It is one year ago they told us that the prospects were bad. One year ago, the illness that took your body in November, with brutal force, extinguished your spark and glow of life. 
One day in heaven - a thousand years on earth. 
We will see you soon again. 
We love you - now, then, and forever. 
/Mommy and Daddy

Our hospital is contributing as a financier in the treatment and research for children with cancer. Our hope is to be able to solve the riddle of childhood cancer.

Currently, our hospital treats 20 children with cancer that do not have health insurance. We hope you can donate, so we can use the money to treath these children and learn to help treat other children.

We want to give a heartily THANK YOU to everyone contributing to us so that we can continue our fight and research against child cancer.

 Together we are strong; together we can make a difference.
